# Risk assessment of retinal vascular occlusion after COVID-19 vaccination

**DOI:** 10.1038/s41541-023-00661-7

**Published:** 2023-05-02

**Authors:** Jing-Xing Li, Yu-Hsun Wang, Henry Bair, Shu-Bai Hsu, Connie Chen, James Cheng-Chung Wei, Chun-Ju Lin

**Affiliations:** 1 Department of General Medicine, China Medical University Hospital, Taichung, Taiwan; 2 School of Medicine, College of Medicine, China Medical University, Taichung, Taiwan; 3 Graduate Institute of Clinical Laboratory Sciences and Medical Biotechnology, National Taiwan University, Taipei, Taiwan; 4 Department of Medical Research, Chung Shan Medical University Hospital, Taichung, Taiwan; 5 Department of Ophthalmology, China Medical University Hospital, Taichung, Taiwan; 6 Byers Eye Institute, Stanford University School of Medicine, Stanford, CA USA; 7 College of Medicine, China Medical University, Taichung, Taiwan; 8 Department of Nursing, China Medical University Hospital, Taichung, Taiwan; 9 Department of Optometry, Chung Shan Medical University, Taichung, Taiwan; 10 Institute of Optometry, Chung Shan Medical University, Taichung, Taiwan; 11 Institute of Medicine, Chung Shan Medical University, Taichung, Taiwan; 12 Department of Allergy, Immunology & Rheumatology, Chung Shan Medical University Hospital, Taichung, Taiwan; 13 Institute of Integrated Medicine, China Medical University, Taichung, Taiwan; 14 Department of Optometry, Asia University, Taichung, Taiwan

**Keywords:** Retinal diseases, Epidemiology

## Abstract

Coronavirus disease 2019 (COVID-19) vaccines are associated with several ocular manifestations. Emerging evidence has been reported; however, the causality between the two is debatable. We aimed to investigate the risk of retinal vascular occlusion after COVID-19 vaccination. This retrospective cohort study used the TriNetX global network and included individuals vaccinated with COVID-19 vaccines between January 2020 and December 2022. We excluded individuals with a history of retinal vascular occlusion or those who used any systemic medication that could potentially affect blood coagulation prior to vaccination. To compare the risk of retinal vascular occlusion, we employed multivariable-adjusted Cox proportional hazards models after performing a 1:1 propensity score matching between the vaccinated and unvaccinated cohorts. Individuals with COVID-19 vaccination had a higher risk of all forms of retinal vascular occlusion in 2 years after vaccination, with an overall hazard ratio of 2.19 (95% confidence interval 2.00–2.39). The cumulative incidence of retinal vascular occlusion was significantly higher in the vaccinated cohort compared to the unvaccinated cohort, 2 years and 12 weeks after vaccination. The risk of retinal vascular occlusion significantly increased during the first 2 weeks after vaccination and persisted for 12 weeks. Additionally, individuals with first and second dose of BNT162b2 and mRNA-1273 had significantly increased risk of retinal vascular occlusion 2 years following vaccination, while no disparity was detected between brand and dose of vaccines. This large multicenter study strengthens the findings of previous cases. Retinal vascular occlusion may not be a coincidental finding after COVID-19 vaccination.

## Introduction

The extremely contagious severe acute respiratory syndrome coronavirus 2 (SARS-CoV-2) is responsible for the coronavirus disease 2019 (COVID-19). Since the end of 2020, many vaccines have been developed, including messenger RNA (mRNA) vaccines (BNT162b2 [Pfizer-BioNTech] and mRNA-1273 [Moderna]), adjuvanted recombinant protein vaccines (Novavax), and adenoviral vector vaccines (ChAdOx1-S vaccine [Oxford/AstraZeneca] and Ad26.COV2.S [Janssen-Johnson&Johnson]). Consequently, several possible complications have been documented due to the increased vaccination rates.

Retinal vein occlusion (RVO) is the second most prevalent cause of visual loss related to retinal vascular diseases, after diabetic retinopathy. RVO is related to thromboembolism caused by vessel compression, vasospasm, or degeneration of vascular walls^1^. Retinal artery occlusion (RAO) is caused by vasospasm, vasculitis, reduced arterial perfusion, and thromboembolism of the retinal arteries originating from the ipsilateral carotid artery, aortic arch, or heart chambers. Based on the location of the occlusion, RAO and RVO can be further classified into central and branch forms. SARS-CoV-2 infection can precipitate retinal vascular events^2,3^. RVO following COVID-19 vaccination is uncommon. However, there is growing literature including case reports on retinal vascular occlusion following vaccination^4–13^. Intriguingly, some studies on retinal vascular occlusion have been related to mRNA COVID-19 vaccination^14,15^; the vaccines implicated include mRNA vaccines, mRNA-1273^10^ and BNT162b2^7,11,16^, as well as the viral vector-based vaccine ChAdOx1^6,12,17^. However, the quality of these data was insufficient to establish a causal relationship between retinal vascular occlusion and COVID-19 vaccination.

This study aimed to determine whether COVID-19 vaccines are related to an increased risk of retinal vascular occlusion and to raise awareness about the probability of retinal vascular events due to an increased thrombotic inflammatory state associated with COVID-19 vaccinations.

## Results

### Patient characteristics and stratified analysis

**Fig. 1:**
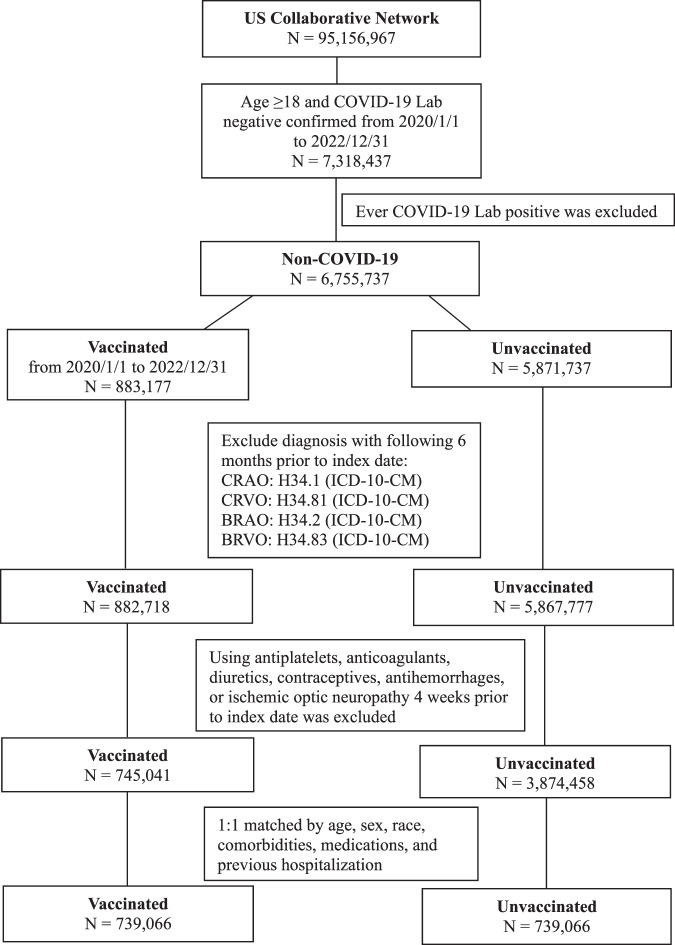
The flowchart of study design to identify vaccinated and unvaccinated cohorts. Access date to the TriNetX database was Feburary 15, 2023.

The TriNetX network collected information on a total of 95,156,967 individuals, of whom 7,318,437 met the inclusion criteria. Figure 1 presents the flowchart of patient selection. After excluding cases with confirmation of COVID-19 diagnosis, 6,755,737 individuals were separated into two cohorts: 883,177 vaccinated and 5,871,737 unvaccinated individuals. In both cohorts, any diagnosis of retinal vascular occlusion six months prior to the index date was excluded. We also considered the effect of systemic medications and excluded cases with the use of any antiplatelets, anticoagulants, diuretics, contraceptives, or antihemorrhages 4 weeks prior to the index date. Ultimately, 745,041 vaccinated and 3,874,458 unvaccinated individuals remained. We matched 739,066 vaccinated cohorts to the unvaccinated cohort at a ratio of 1:1. Table 1 presents the baseline characteristics of the study population. After matching, the average age of the vaccinated group was 52.5 ± 18.5 years, whereas the unvaccinated group was 52.2 ± 18.2 years. There were no differences in any of the variables between the two cohorts. Table 2 reveals the stratified analysis based on age, sex, and race. Individuals aged from 18 to 64 years has increased risk of retinal vascular occlusion except for CRAO.

**Table 1:** Demographic characteristics of patients with and without COVID-19 vaccination.

	Before PSM			After PSM		
	Vaccinated *N* = 745,041	Unvaccinated *N* = 3,874,458	SMD	Vaccinated *N* = 739,066	Unvaccinated *N* = 739,066	SMD
Age	52.6 ± 18.5	46.7 ± 18.1	0.322	52.5 ± 18.5	52.2 ± 18.2	0.016
Sex						
Female	441083 (59.2)	2092041 (54.0)	0.105	436871 (59.1)	434259 (58.8)	0.007
Male	303757 (40.8)	1619285 (41.8)	0.021	301994 (40.9)	269103 (36.4)	0.091
Unknown	201 (0.027)	163132 (4.210)		201 (0.027)	35704 (4.831)	
Race						
White	549172 (73.7)	2437160 (62.9)	0.234	543886 (73.6)	550999 (74.6)	0.022
African American	89896 (12.1)	635883 (16.4)	0.125	89699 (12.1)	91490 (12.4)	0.007
Asian	26491 (3.6)	84507 (2.2)	0.082	26065 (3.5)	25707 (3.5)	0.003
Comorbidities						
Hypertensive diseases	249533 (33.5)	508399 (13.1)	0.496	243707 (33.0)	246040 (33.3)	0.007
Type 2 diabetes mellitus	112966 (15.2)	211428 (5.5)	0.323	108896 (14.7)	105099 (14.2)	0.015
Hyperlipidemia	222993 (29.9)	398604 (10.3)	0.505	217306 (29.4)	220485 (29.8)	0.009
Ischemic heart diseases	73108 (9.8)	122073 (3.2)	0.273	69808 (9.4)	65150 (8.8)	0.022
Cerebrovascular diseases	41250 (5.5)	67952 (1.8)	0.203	38795 (5.2)	34472 (4.7)	0.027
Glaucoma	23249 (3.1)	35391 (0.9)	0.157	22601 (3.1)	14889 (2.0)	0.066
Arterial thromboembolism	6755 (0.9)	7517 (0.2)	0.096	6140 (0.8)	3957 (0.5)	0.036
Venous thromboembolism	16777 (2.3)	24679 (0.6)	0.136	15321 (2.1)	12842 (1.7)	0.025
Pregnancy	19101 (2.6)	85791 (2.2)	0.023	19065 (2.6)	17731 (2.4)	0.012
Overweight and obesity	110743 (14.9)	217725 (5.6)	0.309	106495 (14.4)	104769 (14.2)	0.007
Smoking	64609 (8.7)	172295 (4.4)	0.171	62553 (8.5)	60899 (8.2)	0.008
Medications						
ACEI/ARB	136336 (18.3)	283478 (7.3)	0.333	132620 (17.9)	134004 (18.1)	0.005
Beta-blocker	122425 (16.4)	243745 (6.3)	0.324	118402 (16.0)	116162 (15.7)	0.008
CCB	86635 (11.6)	180981 (4.7)	0.256	84307 (11.4)	80676 (10.9)	0.016
Metformin	55369 (7.4)	112287 (2.9)	0.206	53520 (7.2)	53063 (7.2)	0.002
Lipid lowering agents	164276 (22.0)	329242 (8.5)	0.384	159650 (21.6)	160913 (21.8)	0.004
Corticosteroids	163232 (21.9)	333649 (8.6)	0.376	157965 (21.4)	158061 (21.4)	<0.001
NSAIDs	191038 (25.6)	507084 (13.1)	0.322	186271 (25.2)	188800 (25.5)	0.008
Antipsychotics	61980 (8.3)	137901 (3.6)	0.202	59578 (8.1)	57996 (7.8)	0.008
Previous hospitalization	106573 (14.3)	224481 (5.8)	0.286	102014 (13.8)	97498 (13.2)	0.018

*ACEI* angiotensin converting enzyme inhibitor, *ARB* Angiotensin II receptor blockers, *CCB* Calcium-channel blockers, *N* number, *NSAIDs* non-steroidal anti-inflammatory drugs, *PSM* propensity score matching, *SMD* standardized mean difference.

**Table 2:** Stratified analysis of risk of retinal vascualr occlusion exposed to COVID-19 vaccines compared with unvaccinated individuals in 2 years.

	Number of events		
	Vaccinated	Unvaccinated	HR and 95% Cl
Age 18–64	506,701	506,701	
Retinal vascular occlusion^b^	415	240	1.87 (1.58–2.19)
BRAO	95	63	1.65 (1.19–2.28)
BRVO	174	86	2.21 (1.69–2.87)
CRAO	45	34	1.47 (0.93–2.31)
CRVO	156	78	2.19 (1.65–2.88)
Age ≥ 65	236,804	236,804	
Retinal vascular occlusion^b^	1108	520	2.37 (2.12–2.62)
BRAO	251	140	2.07 (1.67–2.54)
BRVO	443	180	2.70 (2.26–3.21)
CRAO	136	89	1.74 (1.32–2.28)
CRVO	390	169	2.55 (2.12–3.05)
Female	437,682	437,682	
Retinal vascular occlusion^b^	782	359	2.33 (2.05–2.64)
BRAO	168	92	2.02 (1.56–2.61)
BRVO	332	133	2.64 (2.15–3.23)
CRAO	99	58	1.88 (1.34–2.60)
CRVO	272	115	2.54 (2.04–3.16)
Male	302,269	302,269	
Retinal vascular occlusion^b^	731	328	2.40 (2.10–2.73)
BRAO	178	92	2.17 (1.67–2.80)
BRVO	277	104	2.81 (2.23–3.52)
CRAO	87	55	1.69 (1.20–2.37)
CRVO	266	104	2.79 (2.21–3.50)
White	545,223	545,223	
Retinal vascular occlusion^b^	1146	546	2.31 (2.08–2.55)
BRAO	278	149	2.12 (1.72–2.59)
BRVO	465	179	2.79 (2.34–3.31)
CRAO	135	86	1.78 (1.35–2.34)
CRVO	396	175	2.52 (2.10–3.01)
African American	89,833	89,833	
Retinal vascular occlusion^b^	218	118	2.05 (1.63–2.56)
BRAO	40	25	1.85 (1.11–3.07)
BRVO	84	42	2.18 (1.50–3.16)
CRAO	37	19	2.14 (1.22–3.73)
CRVO	83	41	2.28 (1.56–3.31)
Asian	26,080	26,080	
Retinal vascular occlusion^b^	40	16	2.62 (1.46–4.68)
BRAO	<10^a^	<10^a^	1.93 (0.46–8.11)
BRVO	22	<10^a^	2.84 (1.25–6.39)
CRAO	<10^a^	<10^a^	3.69 (0.41–33.00)
CRVO	<10^a^	<10^a^	1.78 (0.64–4.93)

^a^Due to policy of TriNetX, any number less than 10 will be automatically assigned as <10.

^b^The diagnosis of retinal vascular occlusion was determined by its diagnostic codes, rather than by the sum of its subtypes.

*HR* Hazard ratio, *95% CI* 95% confidence interval.

### Brand of vaccines

In the subgroup analysis, the first dose was defined as individuals receiving a single dose of the COVID-19 vaccine, and the second dose was defined as individuals receiving a second dose that was identical to the first dose. Table 3 presents the results of vaccination with various brands of COVID-19 vaccines. The risk of retinal vascular occlusion increased significantly after the first and second doses of BNT162b2 or mRNA-1273 in a 2-year period. The risks were not different between BNT162b2 and mRNA-1273 recipients. Though the risk of retinal vascular occlusion was elevated following the first dose of Ad26.COV2.S, the risk was not significant. Twelve weeks after vaccination of all brands of vaccines, the risk of retinal vascular occlusion increased non-significantly.

**Table 3:** The risk of reintal vascular occlusion significantly increased in individuals receiving the first and second doses of BNT162b2 and mRNA-1273 within 2 years.

	Vaccinated		Unvaccinated		
	Number of events	Incidence (%)	Number of events	Incidence (%)	HR (95% CI)
2 years					
BNT162b2					
First dose	111,491		111,491		
	120	0.036	92	0.021	1.48 (1.12–1.94)
Second dose	96,135		96,135		
	116	0.042	107	0.030	1.36 (1.04–1.77)
mRNA-1273					
First dose	50,382		50,382		
	114	0.064	79	0.044	1.48 (1.10–1.97)
Second dose	47,536		47,536		
	106	0.069	75	0.048	1.50 (1.11–2.02)
Ad26.COV2.S^#^					
First dose	7158		7158		
	<10*	0.140	<10*	0.140	2.35 (0.74–7.39)
Second dose	162		162		
	0	-	0	-	NA
12 weeks					
BNT162b2					
First dose	111,491		111,491		
	40	0.108	23	0.083	1.65 (0.98–2.75)
Second dose	96,135		96,135		
	40	0.121	29	0.111	1.36 (0.84–2.18)
mRNA-1273					
First dose	50,382		50,382		
	32	0.226	22	0.157	1.35 (0.78–2.31)
Second dose	47,536		47,536		
	33	0.223	23	0.158	1.37 (0.80–2.33)
Ad26.COV2.S^#^					
First dose	7158		7158		
	<10*	0.140	<10*	0.140	3.98 (0.44–35.60)
Second dose	162		162		
	0	-	0	-	NA

*HR* Hazard ratio, *NA* not applicable, *95% CI* 95% confidence interval.

*Due to policy of TriNetX, any number less than 10 will be automatically assigned as <10.

^#^A single dose of Ad26.COV2.S was required according to its package insert.

### Risk of retinal vascular occlusion at 2 years and 12 weeks

**Fig. 2:**
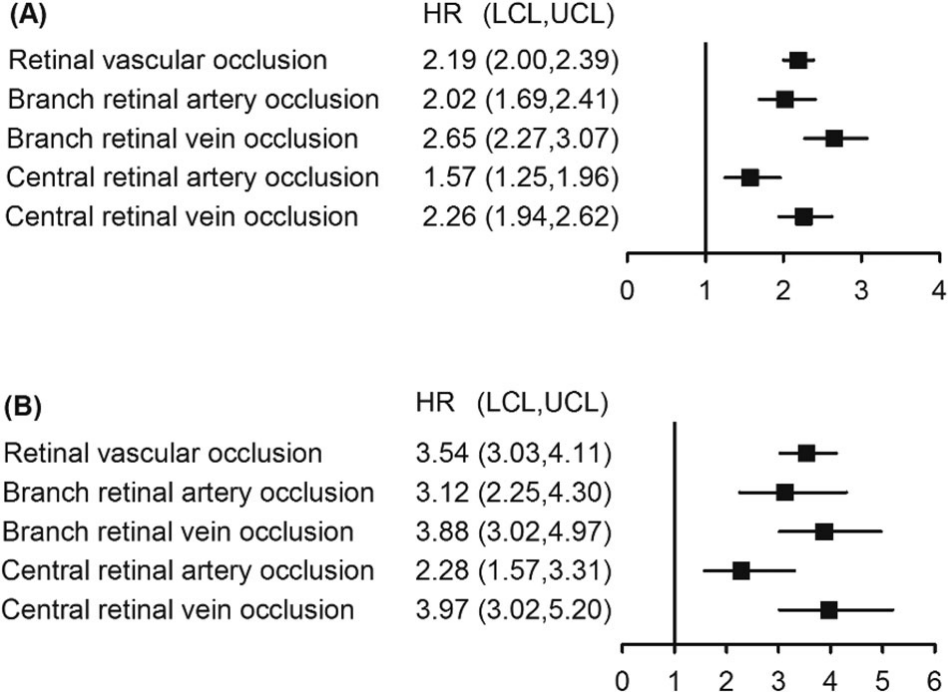
Forest plot of risk of retinal vascular occlusion with 2-year and 12-week follow-up. **A** 2-year, and (**B**) 12-week. *HR* hazard ratio, *LCL* lower confidence limit, *UCL* upper confidence limit, *US* United States.

Figure 2 presents the risk of retinal vascular occlusion at 2 years and 12 weeks after the uptake of COVID-19 vaccines. Supplementary Table 1 shows the original data with number of events and incidence rate. The overall risk of retinal vascular occlusion in the vaccinated cohort was 2.19 times higher than that in the unvaccinated cohort at 2 years (95% Cl 2.00–2.39). Two years after vaccination, the chances of all subtypes (BRAO, BRVO, CRAO and BRVO) of retinal vascular occlusion increased significantly in the vaccinated cohort. The hazards of retinal vascular occlusion and its subtypes were higher within 12 weeks than those at 2 years. Considering the potential acute consequences of COVID-19 vaccinations and its temporary effect, we investigated the bi-weekly incidence of the four forms of retinal vascular occlusion within 12 weeks of COVID-19 vaccination.

### Bi-weekly risk of retinal vascular occlusion in 12 weeks

**Fig. 3:**
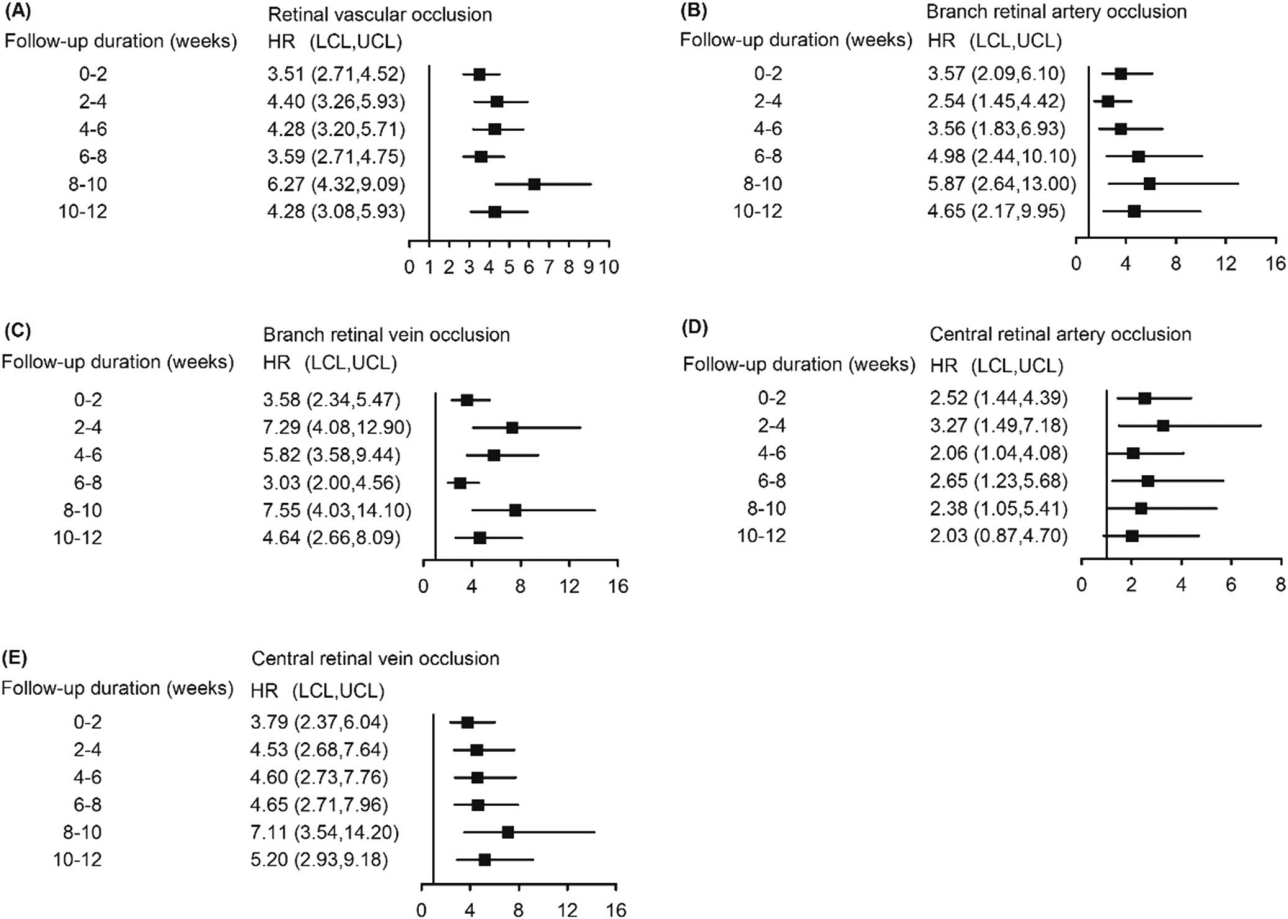
Bi-weekly average hazard ratio of retinal vascular occlusion and its subtypes estimated by cause-specific Cox proportional hazard regression model after COVID-19 vaccination. **A** retinal vascular occlusion, (**B**) branch retinal artery occlusion, (**C**) branch retinal vein occlusion, (**D**) central retinal artery occlusion, and (**E**) central retinal vein occlusion. *HR* hazard ratio, *LCL* lower confidence limit, *UCL* upper confidence limit.

Figure 3 presents the results of the subgroup analysis of bi-weekly risks of retinal vascular occlusion. Supplementary Table 2 provides the original data with number of events and incidence rate. Cox multivariate analysis showed that the risk of retinal vascular occlusion in the vaccinated group was higher than that in the unvaccinated group at within 2 weeks of vaccination, which persisted for 12 weeks. This effect affected all subtypes except CRAO at 10–12 weeks after vaccination.

**Fig. 4:**
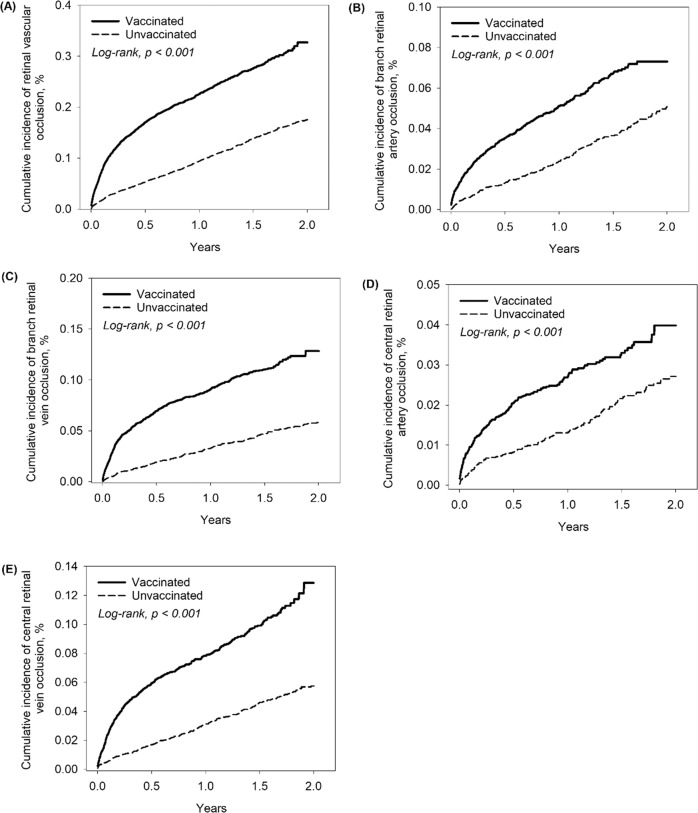
Kaplan–Meier curves showing cumulative incidence of retinal vascular occlusion and its subtypes in 2 years. **A** retinal vascular occlusion, (**B**) branch retinal artery occlusion, (**C**) branch retinal vein occlusion, (**D**) central retinal artery occlusion, and (**E**) central retinal vein occlusion.

Figure 4 demonstrated the results of Kaplan–Meier analysis, which revealed that the cumulative incidence of retinal vascular occlusion and its subtypes were significantly increase in vaccinated than in the unvaccinated cohorts two years after vaccination (log-rank *p* < 0.001). This trend was also observed within 12 weeks after COVID-19 vaccination (log-rank *p* < 0.001) (Supplementary Fig. 1).

### Time course of risk of retinal vascular occlusion

**Fig. 5:**
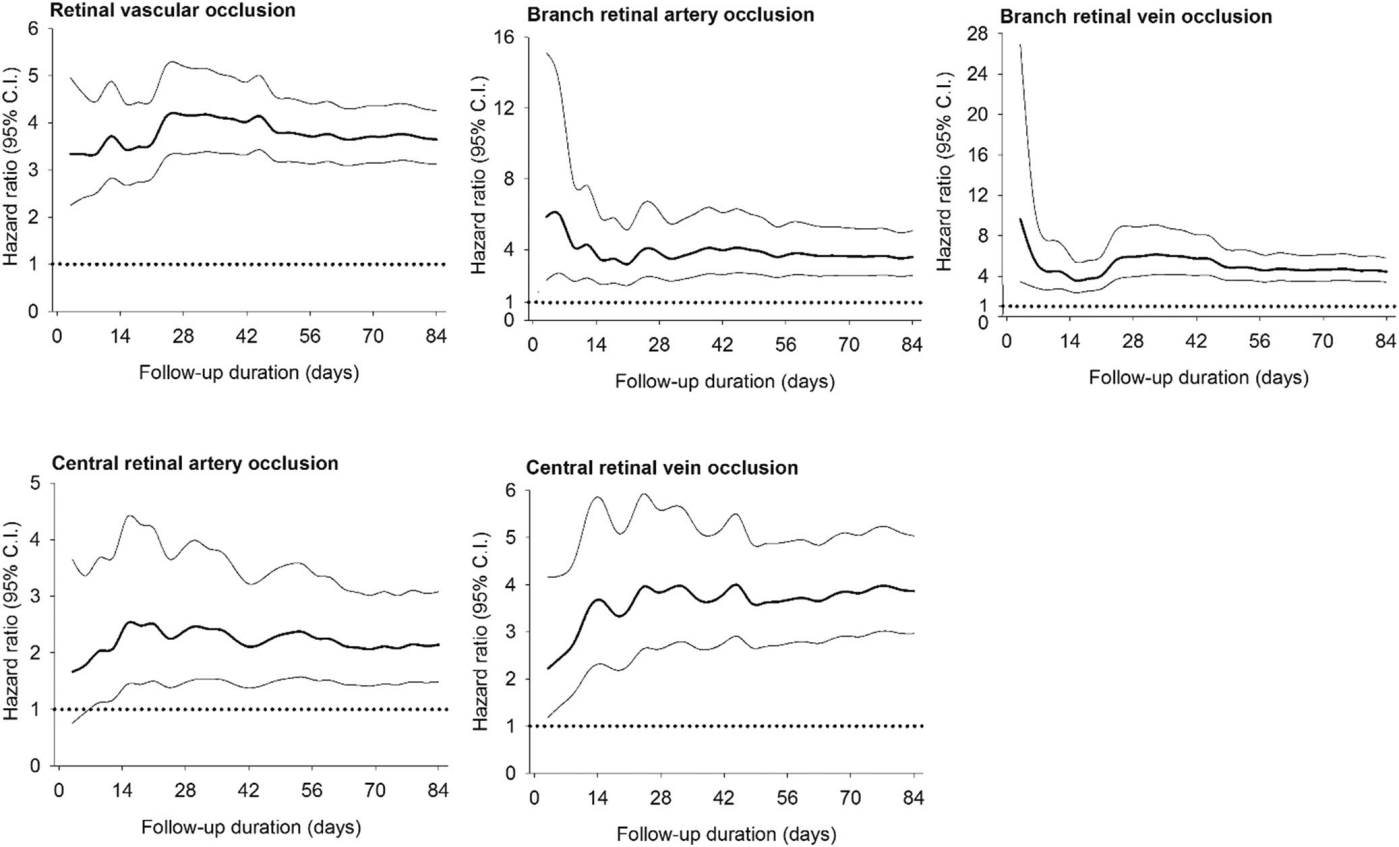
The risks of retinal vascular occlusion and its subtypes significantly elevated following COVID-19 vaccination. Time course of hazard ratios of retinal vascular occlusion and its subtypes following COVID-19 vaccination was demonstrated. Of note, the risk of branch retinal vascular occlusion was extremely high after vaccination.

Figure 5 depicts the time evolution of risks associated with retinal vascular occlusion and its subtypes. The risk of retinal vascular occlusion increased 27 days following vaccination against COVID-19. The risk of branch retinal vascular occlusion was greater at 6 and 3 days for BRAO and BRVO, respectively. In contrast, the probability of central retinal vascular occlusion was greater at 15 and 45 days for CRAO and CRVO. Supplementary Table 3 enumerates hazards of retinal vascular occlusion and its subtypes in 3-day interval after vaccination.

## Discussion

We demonstrated a higher risk and incidence rate of retinal vascular occlusion following COVID-19 vaccination, after adjusting for potential confounding factors^18^. The risk of retinal vascular occlusion, except for CRAO, has been promptly observed in individuals receiving vaccines against SARS-CoV-2. The risk factors for retinal vascular occlusion include diabetes, hypertension, obesity, coronary artery disease, and stroke^19–21^. To ensure the reliability of the results, we appropriately balanced the baseline characteristics in both cohorts before analysis.

The widespread occurrence of microvascular thrombosis in COVID-19 patients have been demonstrated^22^. Vaccination with ChAdOx1 nCoV-19 can result in the rare development of immune thrombotic thrombocytopenia mediated by platelet-activating antibodies against platelet factor 4 (PF4), which clinically mimics autoimmune heparin-induced thrombocytopenia^23^. A large cohort study^24^ showed that the risk of VTE slightly increased 1.10-fold 8–14 days after ChAdOx1 nCoV-19 vaccination but found no difference for individuals who were administered BNT162b2 vaccination; the risk of ATE following ChAdOx1 nCoV-19 and BNT162b2 vaccination increased 1.21-fold and 1.06-fold, respectively.

Thrombosis that manifests before thrombocytopenia is referred to as vaccine-induced immune thrombotic thrombocytopenia (VITT). Two adenoviral vector-based immunizations, ChAdOx1 nCoV-19 and Ad26.COV2.S, have been associated with the development of VITT. VITT cerebral venous sinus thrombosis is predominantly from adenovirus viral vector vaccines. The pathological mechanism of thrombosis has been hypothesized to entail either an innate or adaptive response, involving the activation of B and T cells and CD4 T cells are essential for regulating the production of PF4/heparin-specific antibodies^25^.

VITT is a very rare, life-threatening adverse complication with a 23% overall mortality rate^26^. Certain inflammatory vaccine adjuvants and delivery techniques may induce immune cell recruitment during the VITT. Antibodies that detect platelet-bound PF4 are the cause of VITT. These antibodies are immunoglobulin G (IgG) molecules that activate platelets by binding to platelet FcγIIa with a modest affinity^27^. VITT typically appears as uncommon thromboses (cerebral venous sinus thrombosis and splanchnic vein thrombosis), although it can also manifest as typical thromboses (stroke, pulmonary embolism, and deep vein thrombosis) with severe thrombocytopenia.

Thrombosis with thrombocytopenia syndrome (TTS) is a more general descriptive name for the syndrome of thrombosis and thrombocytopenia of any cause following COVID-19 vaccination. Some individuals with TTS may not have been evaluated for anti-PF4 antibodies; or have causes of thrombosis and thrombocytopenia other than VITT, such as antiphospholipid syndrome, cancer-associated thrombosis and thrombocytopenia, thrombotic thrombocytopenic purpura, or disseminated intravascular coagulation.

A series of 65 individuals with serologically confirmed VITT who repeated functional assays over time found that the functional assays became negative in 74% of individuals, at a median of 15.5 weeks (95% Cl, 5–28 weeks)^28^. VITT plays a fundamental role in retinal vascular disease and may well explain the significantly increased risk of all forms of retinal vascular occlusion in 12 weeks observed in the subgroup analysis. In an examination of the temporal change of the risk of retinal vascular occlusion, which increased significantly shortly after vaccination, especially BRAO and BRVO. The highest hazards of subtypes of retinal vascular occlusion varied. The riskiest period after COVID vaccination for BRAO, BRVO, CRAO, and CRVO was 6, 3, 15, and 45 days, respectively. For BRAO and BRVO, direct embolism may be the preferred mechanism, whereas for CRAO and CRVO, VITT secondary to immunization may be the cause. VITT has a predilection for venous thrombosis in the CNS, splanchnic or adrenal veins, with patients presenting neurologic signs in addition to fever and mild bruising as early as 4–28 and up to 30 days post-COVID-19 vaccination. The relevant literatures on it are extremely limited^29^.

The Netherlands’ Lareb^30^ showed that the incidence rate of VITT and TTS in individuals receiving the ChAdOx1-S vaccine was 7.7 per million vaccinations. Among them, 13.4 per million people who received the first dose and 1.7 per million people who received the second dose. The reported rates of retinal vascular occlusion for Ad26.COV2.S, BNT162b2, and mRNA-1273 per million vaccines were 5.7, 0.05, and 0.2, respectively. The Netherlands Pharmacovigilance Centre Lareb has received three reports of VITT/TTS with BNT162b2 and mRNA-1273; however, the associations are not sufficiently strong. A large international network cohort study^31^ demonstrated a 30% greater risk of thrombocytopenia after a single dose of the ChAdOx1-S vaccine, as well as a trend toward an increased risk of venous TTS after vaccination of Ad26.COV2.S compared with BNT162b2. In this study, though higher risk of retinal vascular occlusion on Ad26.COV2.S recipients was observed in 2-year and 12-week periods, there is no significant increase. Intriguingly, a trend was noted that the risk is more pronounced following immunization with Ad26.COV2.S than BNT162b2 or mRNA-1273.

The SARS-CoV-2 genome encodes ten genes, two-thirds of which are nonstructural. The other one-third of the genome comprises four major structural genes, including spike, envelope, matrix, and nucleocapsid proteins, as well as five auxiliary proteins^32^. Messenger RNA vaccines contain fully functional mRNAs that can be directly translated into the S protein^33,34^. BNT162b2 and mRNA-1273, two mRNA vaccines currently in broad use, are technologically extremely similar. They comprise codon-optimized sequences for effective production of the whole S protein and utilize the actual signal sequence for its biosynthesis. Molecular mimicry of the S protein, which shares sequence homology with human proteins, may play a central role in retinal vascular occlusion^35^.

The global prevalence of RVO, BRVO, and CRVO in individuals aged 30–89 years was 0.77%, 0.64%, and 0.13%^36^. In the United States, the prevalence of RVO, BRVO, and CRVO is 0.7%–0.8%, 0.6%, and 0.1%–0.2%, respectively^37,38^. However, studies on the prevalence of RAO are limited. The current study revealed a strong correlation between vaccination with a mRNA vaccine and retinal vascular occlusion. However, we recommend that individuals without a history of severe allergic reaction to any component of the vaccine be vaccinated to protect against COVID-19, owing to the lack of definite causation between retinal vascular occlusion and vaccinations. Based on the official COVID-19 death reports, it is estimated that vaccinations have prevented 14.4 million excess COVID-19 deaths worldwide between December 2020 and December 2021^39^. Thus, vaccination is the most effective method for preventing the spread of SARS-CoV-2.

The number of reported ophthalmic complications has remained low, and vaccine-related retinal vascular occlusion is very rare, although the number of COVID-19 vaccinations is enormous. As of August 2 2022, 223.04 million people had completed a primary series of COVID-19 vaccines in the US^39^. However, we still suggest that patients on medications that may alter blood osmolarity should be aware of this possibility of adverse effects. Additional research is required to draw a solid conclusion regarding the association between retinal vascular occlusion and COVID-19 vaccines.

### Strengths and weakness

Emerging cases of retinal vascular occlusion in outpatient settings have prompted us to address this concern. However, since this is the first study on this topic, these discoveries may have a significant impact on public health. To ensure the validity of the analysis, we conducted a comprehensive evaluation of confounding factors. However, this study had several limitations. First, since the existence of retinal vascular occlusion was defined by diagnostic codes, the diagnostic accuracy cannot be further confirmed. Second, the HR can be calculated using the TriNetX database; however, the *p*-value is not provided. Third, despite the fact that multiple confounding variables were accounted for, residual confounding variables may still exist and bias the results. Additional clinical investigations are required to validate the efficacy of mRNA vaccination against retinal vascular occlusion. Fourth, underprivileged people are more difficult to seek medical help under COVID-19 pandemic thought they do not have to pay for COVID-19 vaccines. Moreover, retinal vascular occlusion with no or mild symptoms may not be noted. Thus, under-reporting of retinal vascular occlusion and vaccination may bias the study to some extent. Lastly, TriNetX collects patient information only when the patient receives care from one of the participating healthcare organizations. The inclusion of care obtained from other institutions was not possible in this analysis. Loss to follow-up has the potential to distort the distributions of covariates and occurrence of outcomes. In brief, the data should be evaluated critically and cautiously owing to the retrospective nature of this investigation.

This large-scale cohort spanning two years investigate the association between retinal vascular occlusion and COVID-19 vaccination. A 2.19-fold increased risk of retinal vascular occlusion after COVID-19 vaccination was observed. Limited evidence and low frequency of the disease has complicated the establishment of a definitive association between both. The current findings support the conclusions of this case series. This emphasizes the necessity for a thorough study and ophthalmologists to consider the likelihood of retinal vascular occlusion in vulnerable patients following the administration of COVID-19 vaccines. Vaccination is suggested to protect against COVID-19, since the incidence of retinal vascular occlusion remains extremely low.

## Methods

### Study design and participants

This retrospective cohort study was based on data provided by the TriNetX global network, a large and federated research network; numerous renowned studies have employed this database^40–44^. Data for this analysis were restricted to patient data from the United States collected between January 1, 2020 and December 31, 2022, derived from 52 health care organizations. The TriNetX federated network received a waiver from the Western institutional review board since it only aggregated counts and statistical summaries of de-identified information; however, protected health information was not collected, and no study-specific activities were performed in retrospective analyses. The study protocol was approved by institutional review board of Chung Shang Medical University Hospital.

### Outcomes and covariates

The International Classification of Diseases, Tenth Revision, Clinical Modification (ICD-10-CM) codes H34.1 and H34.2, respectively, define central retinal artery occlusion (CRAO) and branch retinal artery occlusion (BRAO). The ICD-10-CM codes H34.81 and H34.83 define central retinal vein occlusion (CRVO) and branch retinal vein occlusion (BRVO), respectively. ICD-10-CM code H34 indicated retinal vascular occlusion. Participants with COVID-19 infection identified by a positive polymerase chain reaction or immunoassay result for immunoglobulin A, G, or M in the plasma or serum were excluded. Participants who received mNRA vaccines BNT162b2 or mRNA-1273 which were documented in electronic medical records during the study period were included. The control group consisted of individuals who had not received any vaccinations. Only individuals with a first-time diagnosis of retinal vascular occlusion during the study period were included in both the case and control groups.

Patients were excluded if a diagnosis of retinal vascular occlusion was made six months before the index date (the earliest date of COVID-19 vaccination) or if they had received antithrombotics, diuretics, oral contraceptives, or antihemorrhagics four weeks prior to the index date. Antithrombotic agents include antiplatelet agents (aspirin, receptor P2Y12 antagonists, and glycoprotein IIb/IIIa [GPIIb/IIIa] inhibitors), anticoagulant (heparin, warfarin, direct oral anticoagulants, and direct thrombin inhibitors), and fibrinolytics (plasminogen activator inhibitors). COVID-19 was identified using TriNetX-provided criteria and ICD-10-CM codes in accordance with the Centers for Disease Control and Prevention coding guidelines. Supplementary Table 4 listed codes of laboratory, diagnosis, and medications adopted on TriNetX platform, as well as ICD-10-CM codes of comorbidities and Anatomical Therapeutic Chemical codes of common-used medications.

### Statistical analysis

We used 1:1 propensity score matching of age, sex, race, comorbidities, medications and previous hospitalization to reduce selection bias and to optimize the variates of the case and control cohorts. The closest propensity scores for the cases and controls were estimated. We used the nearest-neighbor algorithm to derive matched pairs, with values of standardized mean difference <0.1, to indicate a significant difference between the cases and controls. Crude and multivariable-adjusted Cox proportional hazards models were used to compare the risk of outcomes between the cases and controls. The results of the comparisons are presented as HRs and 95% confidence intervals (CIs).

Chi-square (χ^2^) tests were performed to analyze the homogeneity of category variables, including age, sex, race, and comorbidities, between the vaccinated and unvaccinated groups. Comorbidities included hypertensive diseases (ICD-10-CM codes I10–I16), overweight and obesity (ICD-10-CM code E66), type 2 diabetes mellitus (ICD-10-CM code E11), dyslipidemia (ICD-10-CM code E78), cerebrovascular diseases (ICD-10-CM codes I60–I69), ischemic heart diseases (ICD-10-CM code I82), glaucoma (ICD-10-CM code H40), arterial thromboembolism (ATE) (ICD-10-CM code I74), and venous thromboembolism (VTE) (ICD-10-CM code I82). The incidence rates of the retinal vascular occlusion subtypes were calculated for both groups. The 95% confidence interval (CIs) for the risk of retinal vascular occlusion was calculated. The Kaplan–Meier survival curve was plotted to describe the cumulative incidence of retinal vascular occlusion between the two groups, and the differences between the two groups were evaluated using the log-rank test. Statistical significance was set at *p* < 0.05. TriNetX obscures counts in studies with counts less than 10 to protect patient health information by rounding it to the nearest 10. Whenever such rounding occurred in the analysis conducted in this study, it was identified and reported.

### Reporting summary

Further information on research design is available in the Nature Research Reporting Summary linked to this article.

## Supplementary information

Supplementary Info

REPORTING SUMMARY

## Data Availability

Data is available from the TriNetX global network. Requests for data can be sent as logging on TriNetX platform (https://live.trinetx.com/).

## Code availability

Data is available from the TriNetX global network. Requests for data can be sent as logging on TriNetX platform (https://live.trinetx.com/).
