# Supplementary material for: Risk assessment of retinal vascular occlusion after COVID-19 vaccination
Source: NPJ Vaccines. 2023 May 2;8:64. doi: 10.1038/s41541-023-00661-7 (PMC10153772; doi:10.1038/s41541-023-00661-7)

**Supplementary Table 1. Risks of retincal vascular occlusion exposed to vaccinated compared to unvaccinated.**

|  | Vaccinated  N = 739,066 | | Unvaccinated  N = 739,066 | |  |
| --- | --- | --- | --- | --- | --- |
| Follow-up duration | Number of events | Incidence (%) | Number of events | Incidence (%) | HR |
| **2 years** |  |  |  |  |  |
| Retinal vascular occlusion† | 1,506 | 0.204 | 752 | 0.102 | 2.19 (2.00–2.39) |
| BRAO | 347 | 0.047 | 195 | 0.026 | 2.02 (1.69–2.41) |
| BRVO | 606 | 0.082 | 244 | 0.033 | 2.65 (2.27–3.07) |
| CRAO | 185 | 0.025 | 132 | 0.018 | 1.57 (1.25–1.96) |
| CRVO | 540 | 0.073 | 264 | 0.036 | 2.26 (1.94–2.62) |
| **12 weeks** |  |  |  |  |  |
| Retinal vascular occlusion† | 782 | 0.106 | 209 | 0.028 | 3.54 (3.03–4.11) |
| BRAO | 158 | 0.021 | 48 | 0.006 | 3.12 (2.25–4.30) |
| BRVO | 317 | 0.043 | 77 | 0.010 | 3.88 (3.02–4.97) |
| CRAO | 94 | 0.013 | 39 | 0.005 | 2.28 (1.57–3.31) |
| CRVO | 273 | 0.037 | 65 | 0.009 | 3.97 (3.02–5.20) |

^†^ Each disease was retrieved on TriNetX base on ICD-10-CM code, thus the number of retinal vascular occlusion is not merely sum up of its four subtypes.

N, number; BRAO, branch retinal artery occlusion; BRVO, branch retinal vein occlusion; CRAO, central retinal artery occlusion; CRVO, central retinal vein occlusion; HR, hazard ratio.

**Supplementary Table 2. Bi-weekly risks of retinal vascular occlusion exposed to vaccinated compared to unvaccinated 12 weeks following COVID-19 vaccination.**

|  | Vaccinated  N = 741,087 | | Unvaccinated  N = 741,087 | |  |
| --- | --- | --- | --- | --- | --- |
| Follow-up duration | Number of events | Incidence (%) | Number of events | Incidence (%) | HR |
| **0–2 weeks** |  |  |  |  |  |
| Retinal vascular occlusion† | 278 | 0.038 | 75 | 0.010 | 3.51 (2.71–4.52) |
| BRAO | 64 | 0.009 | 17 | 0.002 | 3.57 (2.09–6.10) |
| BRVO | 103 | 0.014 | 27 | 0.004 | 3.58 (2.34–5.47) |
| CRAO | 45 | 0.006 | 17 | 0.002 | 2.52 (1.44–4.39) |
| CRVO | 88 | 0.012 | 22 | 0.003 | 3.79 (2.37–6.04) |
| **2–4 weeks** |  |  |  |  |  |
| Retinal vascular occlusion† | 244 | 0.033 | 52 | 0.007 | 4.40 (3.26–5.93) |
| BRAO | 46 | 0.006 | 17 | 0.002 | 2.54 (1.45–4.42) |
| BRVO | 101 | 0.014 | 13 | 0.002 | 7.29 (4.08–12.90) |
| CRAO | 28 | 0.004 | <10* | 0.001 | 3.27 (1.49–7.18) |
| CRVO | 82 | 0.011 | 17 | 0.002 | 4.53 (2.68–7.64) |
| **4–6 weeks** |  |  |  |  |  |
| Retinal vascular occlusion† | 251 | 0.034 | 56 | 0.008 | 4.28 (3.20–5.71) |
| BRAO | 41 | 0.006 | 11 | 0.001 | 3.56 (1.83–6.93) |
| BRVO | 116 | 0.016 | 19 | 0.003 | 5.82 (3.58–9.44) |
| CRAO | 26 | 0.004 | 12 | 0.002 | 2.06 (1.04–4.08) |
| CRVO | 82 | 0.011 | 17 | 0.002 | 4.60 (2.73–7.76) |
| **6–8 weeks** |  |  |  |  |  |
| Retinal vascular occlusion† | 233 | 0.032 | 62 | 0.008 | 3.59 (2.71–4.75) |
| BRAO | 47 | 0.006 | <10* | 0.001 | 4.98 (2.44–10.10) |
| BRVO | 95 | 0.013 | 30 | 0.004 | 3.03 (2.00–4.56) |
| CRAO | 25 | 0.003 | <10* | 0.001 | 2.65 (1.23–5.68) |
| CRVO | 78 | 0.011 | 16 | 0.002 | 4.65 (2.71–7.96) |
| **8–10 weeks** |  |  |  |  |  |
| Retinal vascular occlusion† | 210 | 0.028 | 32 | 0.004 | 6.27 (4.32–9.09) |
| BRAO | 43 | 0.006 | <10* | 0.001 | 5.87 (2.64–13.00) |
| BRVO | 87 | 0.012 | 11 | 0.001 | 7.55 (4.03–14.10) |
| CRAO | 20 | 0.003 | 10 | 0.001 | 2.38 (1.05–5.41) |
| CRVO | 67 | 0.009 | 10 | 0.001 | 7.11 (3.54–14.20) |
| **10–12 weeks** |  |  |  |  |  |
| Retinal vascular occlusion† | 197 | 0.027 | 44 | 0.006 | 4.28 (3.08–5.93) |
| BRAO | 39 | 0.005 | <10* | 0.001 | 4.65 (2.17–9.95) |
| BRVO | 73 | 0.010 | 15 | 0.002 | 4.64 (2.66–8.09) |
| CRAO | 17 | 0.002 | 10 | 0.001 | 2.03 (0.87–4.70) |
| CRVO | 76 | 0.010 | 14 | 0.002 | 5.20 (2.93–9.18) |

^†^ Each disease was retrieved on TriNetX base on ICD-10-CM code, thus the number of retinal vascular occlusion is not merely sum up of its four subtypes.

* Due to TriNetX’s policy, any number less than 10 will be automatically assigned as <10. While the hazard ratio will be calculated from the real figure.

N, number; BRAO, branch retinal artery occlusion; BRVO, branch retinal vein occlusion; CRAO, central retinal artery occlusion; CRVO, central retinal vein occlusion; HR, hazard ratio.

**Supplementary Table 3.** **Temporal changes of hazard ratios of retinal vascular occlusion and its subtypes following COVID-19 vaccination every 3 days.**

| Follow-up duration (days) | Hazard ratio | | | | |
| --- | --- | --- | --- | --- | --- |
|  | All* | BRAO | BRVO | CRAO | CRVO |
| 3 | 3.341 | 5.863 | **9.625** | 1.666 | 2.221 |
| 6 | 3.336 | **5.924** | 5.71 | 1.797 | 2.474 |
| 9 | 3.37 | 4.144 | 4.447 | 2.035 | 2.814 |
| 12 | 3.72 | 4.279 | 4.427 | 2.079 | 3.49 |
| 15 | 3.441 | 3.426 | 3.588 | **2.517** | 3.66 |
| 18 | 3.494 | 3.488 | 3.746 | 2.479 | 3.355 |
| 21 | 3.557 | 3.178 | 4.073 | 2.507 | 3.499 |
| 24 | 4.12 | 3.934 | 5.544 | 2.26 | 3.953 |
| 27 | **4.189** | 3.973 | 5.911 | 2.342 | 3.845 |
| 30 | 4.157 | 3.508 | 5.995 | 2.466 | 3.919 |
| 33 | 4.181 | 3.626 | 6.148 | 2.42 | 3.958 |
| 36 | 4.113 | 3.863 | 6.017 | 2.4 | 3.7 |
| 39 | 4.082 | 4.101 | 5.942 | 2.229 | 3.645 |
| 42 | 4.024 | 3.968 | 5.742 | 2.105 | 3.809 |
| 45 | 4.142 | 4.114 | 5.692 | 2.166 | **3.991** |
| 48 | 3.831 | 3.989 | 4.962 | 2.289 | 3.611 |
| 51 | 3.793 | 3.852 | 4.859 | 2.35 | 3.618 |
| 54 | 3.752 | 3.58 | 4.818 | 2.366 | 3.638 |
| 57 | 3.714 | 3.758 | 4.559 | 2.254 | 3.693 |
| 60 | 3.766 | 3.759 | 4.728 | 2.244 | 3.711 |
| 63 | 3.666 | 3.649 | 4.647 | 2.123 | 3.651 |
| 66 | 3.659 | 3.653 | 4.575 | 2.092 | 3.776 |
| 69 | 3.707 | 3.631 | 4.637 | 2.063 | 3.854 |
| 72 | 3.708 | 3.61 | 4.668 | 2.112 | 3.822 |
| 75 | 3.755 | 3.615 | 4.715 | 2.078 | 3.93 |
| 78 | 3.746 | 3.644 | 4.554 | 2.148 | 3.973 |
| 81 | 3.68 | 3.513 | 4.583 | 2.12 | 3.895 |
| 84 | 3.651 | 3.583 | 4.45 | 2.142 | 3.863 |

*Retinal vascular occlusion.

The highest figure in each form of retinal vascular occlusion was in bold.

**Supplementary Table 4. Search strategy and definition of study population**

**Diagnosis of COVID-19: Any criteria listed below is positive.**

| **Category** | **Code** | **Laboratory examination** |
| --- | --- | --- |
| **Laboratory** | TNX:9088 | SARS coronavirus 2 and related RNA [Presence] (at least 18 years old at event; lab Result: Negative) |
| **Laboratory** | TNX:9089 | SARS coronavirus 2 IgG IgM Ab [Presence] in Serum or Plasma (at least 18 years old at event; lab Result: Negative) |
| **Laboratory** | UMLS:LNC:94562-6 | SARS-CoV-2 (COVID-19) IgA Ab [Presence] in Serum or Plasma by Immunoassay (at least 18 years old at event; lab Result: Negative) |
| **Laboratory** | UMLS:LNC:94762-2 | SARS-CoV-2 (COVID-19) Ab [Presence] in Serum or Plasma by Immunoassay (at least 18 years old at event; lab Result: Negative) |
| **Laboratory** | UMLS:LNC:94558-4 | SARS-CoV-2 (COVID-19) Ag [Presence] in Respiratory specimen by Rapid immunoassay (at least 18 years old at event; lab Result: Negative) |
| **Laboratory** | UMLS:LNC:95209-3 | SARS-CoV+SARS-CoV-2 (COVID-19) Ag [Presence] in Respiratory specimen by Rapid immunoassay (at least 18 years old at event; lab Result: Negative) |
| **Laboratory** | UMLS:LNC:96119-3 | SARS-CoV-2 (COVID-19) Ag [Presence] in Upper respiratory specimen by Immunoassay (at least 18 years old at event; lab Result: Negative) |

**Type of vaccines:**

| **Type** | **Code** | **Number of patients** |
| --- | --- | --- |
| **BNT** | **91300**, Severe acute respiratory syndrome coronavirus 2 (SARS-CoV-2) (Coronavirus disease [COVID-19]) vaccine, mRNA-LNP, spike protein, preservative free, 30 mcg/0.3mL dosage, diluent reconstituted, for intramuscular use  **0001A** Immunization administration by intramuscular injection of severe acute respiratory syndrome coronavirus 2 (SARS-CoV-2) (Coronavirus disease [COVID-19]) vaccine, mRNA-LNP, spike protein, preservative free, 30 mcg/0.3mL dosage, diluent reconstituted; first dose  **0002A** Immunization administration by intramuscular injection of severe acute respiratory syndrome coronavirus 2 (SARS-CoV-2) (Coronavirus disease [COVID-19]) vaccine, mRNA-LNP, spike protein, preservative free, 30 mcg/0.3mL dosage, diluent reconstituted; second dose | 1,856,508 |
|  | **91305**, Severe acute respiratory syndrome coronavirus 2 (SARS-CoV-2) (coronavirus disease [COVID-19]) vaccine, mRNA-LNP, spike protein, preservative free, 30 mcg/0.3 mL dosage, tris-sucrose formulation, for intramuscular use  **0051A** Immunization administration by intramuscular injection of severe acute respiratory syndrome coronavirus 2 (SARS-CoV-2) (coronavirus disease [COVID-19]) vaccine, mRNA-LNP, spike protein, preservative free, 30 mcg/0.3 mL dosage, tris-sucrose formulation; first dose  **0052A** Immunization administration by intramuscular injection of severe acute respiratory syndrome coronavirus 2 (SARS-CoV-2) (coronavirus disease [COVID-19]) vaccine, mRNA-LNP, spike protein, preservative free, 30 mcg/0.3 mL dosage, tris-sucrose formulation; second dose | 182,648 |
| **Moderna** | **91301,** Severe acute respiratory syndrome coronavirus 2 (SARS-CoV-2) (Coronavirus disease [COVID-19]) vaccine, mRNA-LNP, spike protein, preservative free, 100 mcg/0.5mL dosage, for intramuscular use  **0011A** Immunization administration by intramuscular injection of severe acute respiratory syndrome coronavirus 2 (SARS-CoV-2) (Coronavirus disease [COVID-19]) vaccine, mRNA-LNP, spike protein, preservative free, 100 mcg/0.5mL dosage; first dose  **0012A** Immunization administration by intramuscular injection of severe acute respiratory syndrome coronavirus 2 (SARS-CoV-2) (Coronavirus disease [COVID-19]) vaccine, mRNA-LNP, spike protein, preservative free, 100 mcg/0.5mL dosage; second dose | 325,937 |
|  | **91306,** Severe acute respiratory syndrome coronavirus 2 (SARS-CoV-2) (coronavirus disease [COVID-19]) vaccine, mRNA-LNP, spike protein, preservative free, 50 mcg/0.25 mL dosage, for intramuscular use | 121,337 |
| **AZ** | **91302,** Severe acute respiratory syndrome coronavirus 2 (SARS-CoV-2) (coronavirus disease [COVID-19]) vaccine, DNA, spike protein, chimpanzee adenovirus Oxford 1 (ChAdOx1) vector, preservative free, 5x10^10 viral particles/0.5mL dosage, for intramuscular use  **0021A** Immunization administration by intramuscular injection of severe acute respiratory syndrome coronavirus 2 (SARS-CoV-2) (coronavirus disease [COVID-19]) vaccine, DNA, spike protein, chimpanzee adenovirus Oxford 1 (ChAdOx1) vector, preservative free, 5x10^10 viral particles/0.5mL dosage; first dose  **0022A** Immunization administration by intramuscular injection of severe acute respiratory syndrome coronavirus 2 (SARS-CoV-2) (coronavirus disease [COVID-19]) vaccine, DNA, spike protein, chimpanzee adenovirus Oxford 1 (ChAdOx1) vector, preservative free, 5x10^10 viral particles/0.5mL dosage; second dose | 14 |
| **Johnson & Johnson** | **91303,** Severe acute respiratory syndrome coronavirus 2 (SARS-CoV-2) (coronavirus disease [COVID-19]) vaccine, DNA, spike protein, adenovirus type 26 (Ad26) vector, preservative free, 5x10^10 viral particles/0.5mL dosage, for intramuscular use  **0031A** Immunization administration by intramuscular injection of severe acute respiratory syndrome coronavirus 2 (SARS-CoV-2) (coronavirus disease [COVID-19]) vaccine, DNA, spike protein, adenovirus type 26 (Ad26) vector, preservative free, 5x10^10 viral particles/0.5mL dosage, single dose  **0034A** Immunization administration by intramuscular injection of severe acute respiratory syndrome coronavirus 2 (SARS-CoV-2) (coronavirus disease [COVID-19]) vaccine, DNA, spike protein, adenovirus type 26 (Ad26) vector, preservative free, 5x10^10 viral particles/0.5 mL dosage; booster dose | 52,505 |
| **Generic** | **213,** SARS-CoV-2 (COVID-19) Vaccine  **2468231,**  SARS-CoV-2 (COVID-19) vaccine, mRNA spike protein  **2479831,** SARS-COV-2 (COVID-19) vaccine, vector non-replicating | 2,449,677  2,091,764  72,668 |

**Outcomes:**

| Category | Code | Disease |
| --- | --- | --- |
| Diagnosis | UMLS:ICD10CM:H34.2 | Branch retinal artery occlusions |
| Diagnosis | UMLS:ICD10CM:H34.83 | Branch retinal vein occlusion |
| Diagnosis | UMLS:ICD10CM:H34.1 | Central retinal artery occlusion |
| Diagnosis | UMLS:ICD10CM:H34.81 | Central retinal vein occlusion |

**Disease excluded in this study:**

| Category | Code | Disease |
| --- | --- | --- |
| Diagnosis | UMLS:ICD10CM: H47.01 | AION: Ischemic optic neuropathy |

**Medications excluded in this study:**

| Category | Code | Generic name |
| --- | --- | --- |
| Medication | NLM:ATC:B01A | Antithrombotic agents |
| Medication | NLM:ATC:B01AA | Vitamin K antagonists |
| Medication | NLM:ATC:B01AB | Heparin group |
| Medication | NLM:ATC:B01AC | Platelet aggregation inhibitors excl. heparin |
| Medication | NLM:ATC:B01AD | Enzymes |
| Medication | NLM:ATC:B01AE | Direct thrombin inhibitors |
| Medication | NLM:ATC:B01AF | Direct factor Xa inhibitors |
| Medication | NLM:RXNORM:11289 | Warfarin |
| Medication | NLM:RXNORM:1114195 | Rivaroxaban |
| Medication | NLM:RXNORM:1037042 | Dabigatran etexilate |
| Medication | NLM:RXNORM:1599538 | Edoxaban |
| Medication | NLM:RXNORM:1364430 | Apixaban |
| Medication | NLM:ATC:B01AE | Direct thrombin inhibitors |
| Medication | NLM:RXNORM:1191 | Aspirin |
| Medication | NLM:RXNORM:10594 | Ticlopidine |
| Medication | NLM:RXNORM:613391 | Prasugrel |
| Medication | NLM:RXNORM:32968 | Clopidogrel |
| Medication | NLM:RXNORM:1116632 | Ticagrelor |
| Medication | NLM:RXNORM:1656052 | Cangrelor |
| Medication | NLM:RXNORM:83929 | Abciximab |
| Medication | NLM:RXNORM:73137 | Tirofiban |
| Medication | NLM:ATC:C03 | Diuretics |
| Medication | NLM:ATC:G03A | Hormonal contraceptives for systemic use |
| Medication | NLM:ATC:B02 | Antihemorrhagics |

**Comorbidities and Medications:**

| **Codes** | **Comorbidities/Medications** |
| --- | --- |
| ICD-10-CM code |  |
| I10–I16 | Hypertensive diseases |
| E66 | Overweight and obesity |
| E11 | Type 2 diabetes mellitus |
| E78 | Disorders of lipoprotein metabolism and other lipidemias |
| I20–I25 | Ischemic heart diseases |
| I60–I69 | Cerebrovascular diseases |
| H40 | Glaucoma |
| I74 | Arterial embolism and thrombosis |
| I82 | Other venous embolism and thrombosis |
| Z34 | Pregnancy (encounter for supervision of normal pregnancy) |
| F17 | Smoking (nicotine dependence) |
| ATC code |  |
| C09 | ACEi/ARB (agents acting on the renin-angiotensin system) |
| C07 | Beta-blocker (beta blocking agents) |
| C08 | Calcium-channel blockers |
| A10BA02 | Metformin |
| C10 | Lipid modifying agents |
| C05AA | Corticosteroids |
| M01A | NSAIDs (anti-inflammatory and antirheumatic products, non-steroids) |
| N05A | Antipsychotics |

ATC, Anatomical Therapeutic Chemical; ICD-10-CM, International Classification of Diseases, Tenth Revision, Clinical Modification.

**Supplementary Figure 1. Kaplan–Meier curves showing cumulative incidence of retinal vascular occlusion and its subtypes in 12 weeks.**


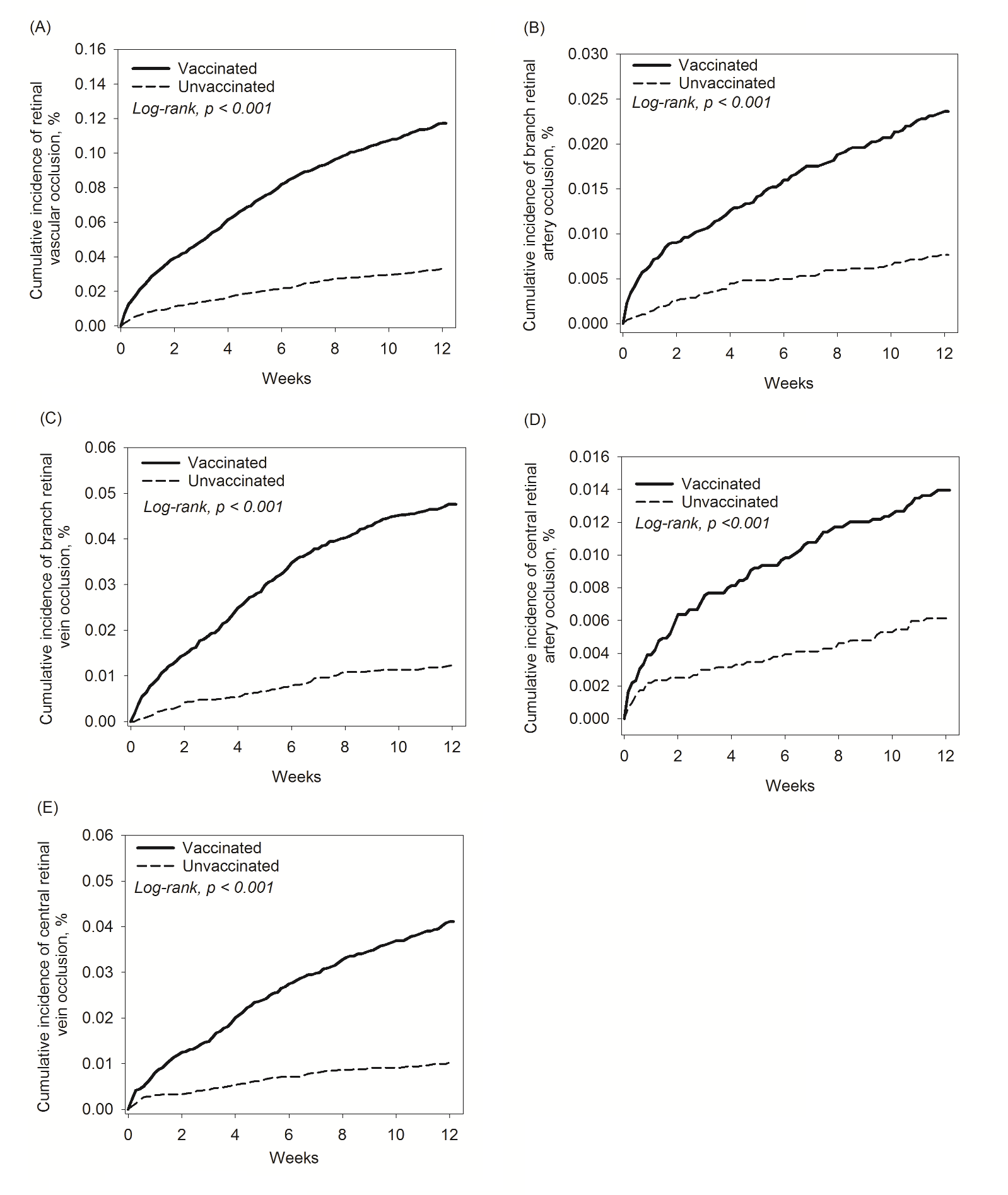

Supplement: Supplementary file 1 — Supplementary Info [file 41541_2023_661_MOESM1_ESM.docx]
