# Supplementary material for: Risk assessment of retinal vascular occlusion after COVID-19 vaccination
Source: NPJ Vaccines. 2023 May 2;8:64. doi: 10.1038/s41541-023-00661-7 (PMC10153772; doi:10.1038/s41541-023-00661-7)
Supplement: Supplementary file 2 — REPORTING SUMMARY [file 41541_2023_661_MOESM2_ESM.pdf]

nature portfolio

Corresponding author(s): JCCW and CJL

Last updated by author(s): Mar 22, 2023

Reporting Summary

Nature Portfolio wishes to improve the reproducibility of the work that we publish. This form provides structure for consistency and transparency in reporting. For further information on Nature Portfolio policies, see our Editorial Policies and the Editorial Policy Checklist.

Statistics

For all statistical analyses, confirm that the following items are present in the figure legend, table legend, main text, or Methods section.

n/a	Confirmed	
☐	☒	The exact sample size (*n*) for each experimental group/condition, given as a discrete number and unit of measurement
☐	☒	A statement on whether measurements were taken from distinct samples or whether the same sample was measured repeatedly
☒	☐	The statistical test(s) used AND whether they are one- or two-sided<br>*Only common tests should be described solely by name; describe more complex techniques in the Methods section.*
☐	☒	A description of all covariates tested
☒	☐	A description of any assumptions or corrections, such as tests of normality and adjustment for multiple comparisons
☐	☒	A full description of the statistical parameters including central tendency (e.g. means) or other basic estimates (e.g. regression coefficient) AND variation (e.g. standard deviation) or associated estimates of uncertainty (e.g. confidence intervals)
☐	☒	For null hypothesis testing, the test statistic (e.g. *F*, *t*, *r*) with confidence intervals, effect sizes, degrees of freedom and *P* value noted<br>*Give P values as exact values whenever suitable.*
☒	☐	For Bayesian analysis, information on the choice of priors and Markov chain Monte Carlo settings
☒	☐	For hierarchical and complex designs, identification of the appropriate level for tests and full reporting of outcomes
☒	☐	Estimates of effect sizes (e.g. Cohen's *d*, Pearson's *r*), indicating how they were calculated

Our web collection on statistics for biologists contains articles on many of the points above.

Software and code

Policy information about availability of computer code

Data collection	No software was used.
Data analysis	No software was used. All data was anaylzed on TriNetX platform.

For manuscripts utilizing custom algorithms or software that are central to the research but not yet described in published literature, software must be made available to editors and reviewers. We strongly encourage code deposition in a community repository (e.g. GitHub). See the Nature Portfolio guidelines for submitting code & software for further information.

Data

Policy information about availability of data

All manuscripts must include a data availability statement. This statement should provide the following information, where applicable:

- Accession codes, unique identifiers, or web links for publicly available datasets
- A description of any restrictions on data availability
- For clinical datasets or third party data, please ensure that the statement adheres to our policy

Data is available from the TriNetX global network. Requests for data can be sent as logging on TriNetX platform (https://live.trinetx.com/).

Human research participants

Policy information about studies involving human research participants and Sex and Gender in Research.

Reporting on sex and gender	Both sexes were included in this study according to the electronic medical records.
Population characteristics	Characteristics included age, sex, race, comorbidities (hypertension, type 2 diabetes, hyperlipidemia, ischemic heart disease, cerebrovascular disease, glaucoma, arterial/venous thromboembolism, pregnancy, obesity and smoking), medications and previous hospitalization.
Recruitment	The TriNetX network collected information on a total of 95,156,967 individuals, of whom 7,318,437 met the inclusion criteria. After excluding cases with confirmation of COVID-19 diagnosis, 6,755,737 individuals were separated into two cohorts: 883,177 vaccinated and 5,871,737 unvaccinated individuals. In both cohorts, any diagnosis of retinal vascular occlusion six months prior to the index date was excluded. We also considered the effect of systemic medications and excluded cases with the use of any antiplatelets, anticoagulants, diuretics, contraceptives, or antihemorrhages 4 weeks prior to the index date. Ultimately, 745,041 vaccinated and 3,874,458 unvaccinated individuals remained.
Ethics oversight	Institutional review board of Chung Shan Medical University Hospital

Note that full information on the approval of the study protocol must also be provided in the manuscript.

Field-specific reporting

Please select the one below that is the best fit for your research. If you are not sure, read the appropriate sections before making your selection.

☒ Life sciences ☐ Behavioural & social sciences ☐ Ecological, evolutionary & environmental sciences

For a reference copy of the document with all sections, see nature.com/documents/nr-reporting-summary-flat.pdf

Life sciences study design

All studies must disclose on these points even when the disclosure is negative.

Sample size	The TriNetX network collected information on a total of 95,156,967 individuals, of whom 7,318,437 met the inclusion criteria. After excluding cases with confirmation of COVID-19 diagnosis, 6,755,737 individuals were separated into two cohorts: 883,177 vaccinated and 5,871,737 unvaccinated individuals. After exclusion, 745,041 vaccinated and 3,874,458 unvaccinated individuals remained. We did the propensity-score matching of 739,066 vaccinated cohorts to the unvaccinated cohort at a ratio of 1:1.
Data exclusions	Individuals with a history of retinal vascular occlusion within six months prior to vaccination or use of any systemic medication that may alter blood coagulation within four weeks prior to vaccination were excluded.
Replication	TriNetX used electronic healthcare records on dynamic basis. The statistical population is changing with access time. However, the results were consistent with good reproducibility in this study.
Randomization	No randomization process is adopted in this study. Before analysis, we used propensity-score matching of variates between case and control group to minimize confounding factors.
Blinding	This is retrospective cohort study, no randomization or blinding was indicated.

Reporting for specific materials, systems and methods

We require information from authors about some types of materials, experimental systems and methods used in many studies. Here, indicate whether each material, system or method listed is relevant to your study. If you are not sure if a list item applies to your research, read the appropriate section before selecting a response.

Materials & experimental systems

n/a	Involved in the study
☒	☐ Antibodies
☒	☐ Eukaryotic cell lines
☒	☐ Palaeontology and archaeology
☒	☐ Animals and other organisms
☒	☐ Clinical data
☒	☐ Dual use research of concern

Methods

n/a	Involved in the study
☒	☐ ChIP-seq
☒	☐ Flow cytometry
☒	☐ MRI-based neuroimaging
